# Prevalence of Anxiety and Depression among Outpatients with Type 2 Diabetes in the Mexican Population

**DOI:** 10.1371/journal.pone.0036887

**Published:** 2012-05-18

**Authors:** Carlos Tovilla-Zárate, Isela Juárez-Rojop, Yesenia Peralta Jimenez, María Antonia Jiménez, Silvia Vázquez, Deysi Bermúdez-Ocaña, Teresa Ramón-Frías, Alma D. Genis Mendoza, Sherezada Pool García, Lilia López Narváez

**Affiliations:** 1 Universidad Juárez Autónoma de Tabasco, División Académica Multidisciplinaria de Comalcalco, Comalcalco, Tabasco, México; 2 Universidad Juárez Autónoma de Tabasco, División Académica de Ciencias de la Salud, Villahermosa, Tabasco, México; 3 Servicios de Atención Psiquiátrica, Secretaría de Salud. México D. F., México; 4 Hospital General de Comalcalco, Tabasco. Secretaría de Salud, Comalcalco, Tabasco, México; 5 Centro de Investigación Genómica, Comalcalco, Tabasco, México; Vanderbilt University, United States of America

## Abstract

**Background:**

Depression and anxiety are common in diabetic patients; however, in recent years the frequency of these symptoms has markedly increased worldwide. Therefore, it is necessary to establish the frequency and factors associated with depression and anxiety, since they can be responsible for premature morbidity, mortality, risk of developing comorbidities, complications, suffering of patients, as well as escalation of costs. We studied the frequency of depression and anxiety in Mexican outpatients with type 2 diabetes and identified the risk factors for depression and anxiety.

**Methods and Findings:**

We performed a study in 820 patients with type 2 diabetes. The prevalence of depression and anxiety was estimated using the Hamilton Depression Rating Scale and the Hamilton Anxiety Rating Scale, respectively. We calculated the proportions for depression and anxiety and, after adjusting for confounding variables, we performed multivariate analysis using multiple logistic regressions to evaluate the combined effect of the various factors associated with anxiety and depression among persons with type 2 diabetes. The rates for depression and anxiety were 48.27% (95% CI: 44.48–52.06) and 55.10% (95% CI: 51.44–58.93), respectively. Occupation and complications in diabetes were the factors associated with anxiety, whereas glucose level and complications in diabetes were associated with depression. Complications in diabetes was a factor common to depression and anxiety (p<0.0001; OR 1.79, 95% CI 1.29–2.4).

**Conclusions:**

Our findings demonstrate that a large proportion of diabetic patients present depression and/or anxiety. We also identified a significant association between complications in diabetes with depression and anxiety. Interventions are necessary to hinder the appearance of complications in diabetes and in consequence prevent depression and anxiety.

## Introduction

The global prevalence of diabetes is continuously rising. It is estimated that almost 285 million persons are currently suffering from diabetes worldwide, and the number is expected to rise to 438 million by the year 2030; more than 70% of these individuals reside in developing countries [1]. The literature reports that patients with diabetes are almost twice as likely to suffer from depression and anxiety as the general population [2], [3]. Recently, a biologic mechanism has been suggested that associates both depression and diabetes with deregulated and overactive hypothalamic-pituitary-adrenal axis activity [4], [5]. Depression as a chronic psychological stress is associated with sub-clinical hypercortisolism secondary to the activation of the hypothalamic-pituitary-adrenal axis [5]. Cortisol is a counterregulatory hormone and its prolonged exposure induces visceral adiposity, insulin resistance, dyslipidemia, and hypertension (all metabolic precursors to type 2 diabetes). This hormone stimulates the sympathetic nervous system, increases inflammatory and platelet aggregation responses, and decreases insulin sensitivity [1], [5], [6], [7]. This suggests that increased cortisol in diabetes can be considered a risk factor for the development and presence of depression and anxiety. These symptoms can lead to consequences in the life of patients, given that emotional problems may influence patient adherence to lifestyle and treatment recommendations. As a result, decreased quality of life, impaired self-care behavior, and poorer glycemic control may ensue and contribute to increase health care costs [2], [8].

Depression is associated with a wide range of negative consequences, including significant worsening of comorbid medical conditions, high mortality risk related to suicide, and socioeconomic burden resulting from functional impairment [9], [10]. Recently, both diabetes and depression have been associated with premature morbidity and mortality, and when these conditions coexist, the risk for developing comorbidities, complications, suffering of patients, and associated costs escalates [11], [12]. Therefore, it is relevant to establish the diagnoses of depression and anxiety in the diabetic patient. The presence of undiagnosed anxiety and depression among persons with this condition is a cause of concern since these symptoms hinder the initiation of treatment and allows frustration to build up in patients, thereby contributing to poor clinical outcomes [1].

In Mexico, diabetes is a common disorder. The prevalence in the general population is 7% [13]; 6.5% in males and 7.3% in females. The frequency of this disease is on the rise. In 1993, its prevalence was 4.6%; nevertheless, in the year 2000, it increased to 5.8%, and 7% in 2006 [13]. Among the Mexican general population, anxiety exhibits a frequency of 14.3%, whereas for depression it amounts to 9.1% [14]. These frequencies are similar to the rates observed in the United States [15]. However, several reports in Mexico have shown that the prevalence of depression in diabetic patients can be 46%, 48.3% and 63% [16], [17], [18]. In contrast, there are no reports in the literature evaluating anxiety in the Mexican population.

Various studies to assess anxiety and depression and their associated factors among diabetic patients have been undertaken in many developed countries. For example, a sectional study in the United Kingdom found that almost one-third of diabetic persons suffer from anxiety and one-fourth from depression. This outcome is in agreement with the results in other similar studies [1], [19], [20], [21], [22]. However, only three studies have reported an association between depression and diabetes in the Mexican population [16], [17], [18], with the concomitant limitation of having been carried out in small samples. Hence, in this work, we determined the frequency of depression and anxiety in outpatients with type 2 diabetes in the southeastern part of Mexico. This study also provides socio-epidemiologic findings of the participants, as well as risk factors for depression and anxiety underlying this condition.

## Results

### Prevalence of Anxiety and Depression

From 827 participants only 704 that complied with the inclusion criteria were included. From this sample, 48.27% (95% CI: 44.48–52.06) were positive for depression and 55.10% (95% CI: 51.44–58.93) for anxiety.

### Descriptive Characteristics

In this sample, the larger group was formed of females (55.8%). Most of them were married (71.4%) and mainly housewives (39.0%). The socio-demographic characteristics of the participants are summarized in Table 1. Mean age was 47.39±12.79 with a range of 18–79 years old. The mean level of education was 8.81±4.91 years. Thirty-eight patients did not receive formal education or were illiterate. The mean body mass index was 28.63±5.49 with a range of 17–47. The average glucose level was 175.46±74.37 mg/dl, with a range of 70–500 mg/dl. The mean systolic BP was 120.00±13.14 (range 90–180); the corresponding average diastolic BP was 80.14±10.84 (range 60–130). When we analyzed anxiety the average score was 17.97±11.39 (range 1–39), whereas the mean score for depression was 18.58±11.06, with a range of 1–49.

**Table 1:** Socio-epidemiologic characteristics and gender differences of patients with type 2 diabetes.

Characteristics		Number	Percent
Gender	Male	310	44.2
	Female	392	55.8
Marital status	Married	501	71.4
	Single	100	14.2
	Widowed	68	9.7
	Separated/divorced	33	4.7
Occupation	Unemployed	54	7.7
	Housewife	274	39.0
	Student	41	5.8
	Full-time job	142	20.2
	Half-time job	191	27.2
Age (in years)	Up to 50	410	41.7
	>50	292	58.3
Education	Up to 6 years of schooling	300	42.7
	>6 years of schooling	402	57.3
Body mass index	Up to 25	239	34.0
	>25	463	66.0
Glucose level (mg/dl)	Up to 120	131	18.6
	>120	572	81.4
Use of substances	Yes	325	46.3
	No	378	53.7
Complications	Yes	260	36.9
	No	443	63.1
Anxiety	With anxiety	388	52.9
	Without anxiety	314	47.1
Depression	With depression	335	47.7
	Without depression	368	52.3

### Association of Dependent Variables with Depression and Anxiety

Table 2 summarizes the results of univariate and multivariate analyses for associations with anxiety. The factors that showed a significant association with anxiety were occupation and presence of complications. On the other hand, the factors significantly associated with depression were glucose level and presence of complications (see Table 3).

**Table 2:** Association between clinical characteristics and anxiety in patients with type 2 diabetes.

		WithAnxiety	Without Anxiety	p-value	OR (95% CI)	p-value	AOR (95% CI)*
Gender	Male	165	145	0.33	0.86 (0.63–1.16)	0.17	1.25 (0.90–1.75)
	Female	223	169				
Marital status	Married	274	227	0.77		0.70	1.03 (0.86–1.23)
	Single	60	40				
	Widowed	36	32				
	Separated/divorced	13	15				
Occupation	Unemployed	32	22	**0.04**		0.14	0.91 (0.81–1.03)
	Housewife	157	117				
	Student	26	15				
	Full-time job	85	57				
	Half-time job	88	103				
Age (in years)	Up to 50	230	180	0.56	1.09 (0.80–1.97)	0.55	0.91 (0.67–1.23)
	>50	158	135				
Education	Up to 6 years of schooling	169	219	0.78	1.04 (0.77–1.40)	0.16	1.25 (0.91–1.71)
	>6 years of schooling	134	181				
Body mass index	Up to 25	131	108	0.88	097 (0.71–1.33)	0.23	1.19 (0.88–1.61)
	>25	257	207				
Glucose level (mg/dl)	Up to 120	67	64	0.30	0.81 (0.55–1.19)	**0.03**	1.44 (1.03–2.01)
	>120	321	251				
Use of substances	Yes	187	138	0.24	1.19 (0.88–1.60)	0.18	0.80 (0.58–1.11)
	No	201	177				
Complications	yes	167	93	**0.0001**	1.80 (1.31–2.47)	**0.003**	0.62 (0.45–0.85)
	No	221	222				

* Logistic regression model adjusted for all variables in the table.

CI: confidence interval; OR: odds ratio; AOR, adjusted odds ratio.

**Table 3:** Association between clinical characteristics and depression in patients with type 2 diabetes.

		With depression	Without depression	p-value	OR (95% CI)	p-value	AOR (95% CI)*
Gender	Male	139	171	0.17	0.81 (0.60–1.09)	0.07	1.36 (0.96–1.93)
	Female	196	196				
Marital status	Married	234	267	0.56		0.43	1.07 (0.89–1.28)
	Single	51	49				
	Widowed	31	37				
	Separated/divorced	19	14				
Occupation	Unemployed	21	33	0.25		0.83	1.01 (0.89–1.14)
	Housewife	135	139				
	Student	21	20				
	Full-time job	75	67				
	Half-time job	21	33				
Age (in years)	Up to 50	195	215	0.95	0.99 (0.73–1.33)	0.63	0.91 (0.65–1.29)
	>50	140	153				
Education	Up to 6 years of schooling	146	157	0.80	1.03 (0.76–1.40)	0.78	1.04 (0.74–1.47)
	>6 years of schooling	189	211				
Body mass index	Up to 25	122	117	0.19	1.22 (0.89–1.68)	0.29	0.84 (0.61–1.16)
	>25	213	251				
Glucose level (mg/dl)	Up to 120	49	82	**0.009**	0.59 (0.40–0.87)	**0.021**	1.60 (1.07–2.38)
	>120	286	286				
Use of substances	Yes	164	161	0.16	1.23 (0.91–1.06)	0.08	0.74 (0.53–1.03)
	No	171	207				
Complications	yes	140	120	**0.01**	1.40 (1.06–1.97)	0.67	0.67 (0.48–0.93)
	No	195	243				

* Logistic regression model adjusted for all variables in the table.

CI: confidence interval; OR: odds ratio; AOR, adjusted odds ratio.

### Multivariate Analysis

Finally, we looked for an association of independent factors with anxiety and depression. As a result, we encountered that patients with a history of complications with diabetes were significantly associated with anxiety (p<0.0001; OR 1.79, 95% CI 1.29–2.4). When this value was adjusted for confounding variables, the result was similar (AOR 1.74, 95% CI 1.25–2.41). Likewise, the factors housewife (AOR 1.52, 95% CI 1.01–2.28), student (AOR 2.34, 95% CI 1.14–4.80) and half-time employee (AOR 1.69, 95% CI 1.08–2.66) presented associations.

## Discussion

We estimated the prevalence of depression and anxiety in a type 2 diabetic population in the state of Tabasco in southern Mexico. This study also showed that glucose levels and complications with diabetes were significantly associated with depression; the factors to have or not have occupation and complications in diabetes were associated with anxiety.

It is important to acknowledge the prevalence of depression and/or anxiety in diabetic patients since the development of co-morbid anxiety and/or depression in people with this condition not only leads to increase disease severity, complications, work disability, and poor quality of life, but is also associated with increased used of medical services and substantially higher health care costs [1], [11], [12], [23]. Currently, to recognize depression in diabetic patients is not a general practice.

In this study, the prevalence of depression was estimated at 48.27%. In the literature there are three studies on the Mexican population that have analyzed this prevalence [16], [17], [18]. And although these studies were carried out in different regions of Mexico, they exhibited similar frequencies, viz., 46%, 48.3% and 63%. However, sample sizes were small (n = 79,186 and 450, respectively). In the present study, sample size was increased to 704 diabetic patients. In addition, we analyzed the frequency of anxiety in these patients. To our knowledge, this is the first study analyzing both depression and anxiety in the Mexican population. Frequently, a higher prevalence of depression has been reported in developed countries [24], [25], [26]. However, we found a similar prevalence to the one reported in those studies. In fact, recent studies have shown a similar prevalence in under-developed countries [1], [27].

The frequency of anxiety in our study was 55.10%. In the literature, it has been frequently observed that anxiety has more prevalence than depression. Besides, a higher prevalence of anxiety is present in people suffering from chronic diseases [19], [28], [29]. In the Mexican population, depression causes on average 25.51 days of disability leaves of absence and anxiety 9.53 days on average [30]. Hence, alternative mechanisms are necessary for the prevention of depression and anxiety in diabetic patients.

We observed that the development of complications in diabetes is a common factor associated with depression and anxiety; this is consistent with the results in other studies reporting an association with duration of diabetes [1], [31], [32]. In this respect, the duration of diabetes is associated with the development of depression. Increased duration of the disease is known to significantly increase the risk for developing diabetic complications and health care expenditures, as a result such patients are more prone to develop psychological illnesses.

Some limitations can be identified in this study. The patients with diabetes were only analyzed once and not at different times within the duration of the study. The second limitation is that the results were similar to studies using a self-report instrument to measure depression and anxiety. In this case, dependent and independent variables could possibly be carrying intrinsic respondent biases and measurement errors. Third, we did not utilize a scale for diagnosing depression in the general population; our main interest was to identify the presence of depression and anxiety symptoms in diabetic patients. Fourth, this is not a random sample, and it might not be epidemiologically valid, however, it provides an estimation of the prevalence of depression and anxiety in patients with type 2 diabetes in Tabasco, Mexico. Hence, caution must be taken when generalizing these results to the entire Mexican population.

In conclusion, this study shows a high prevalence of depression and anxiety in a large sample of the Mexican population suffering from diabetes. Besides, the development of complications in diabetes was the principal factor associated with depression and anxiety in these patients. Therefore, we suggest that it is necessary to test diabetic patients for depression and anxiety using psychiatric diagnosis to prevent the appearance of these symptoms. Finally, more comprehensive studies are necessary to determine conclusively the pathophysiological mechanisms present in this disease.

## Methods

A total of 820 outpatients with type 2 diabetes were enrolled in this study. Recruitment took place from January 2011 to September 2011. This is a multi-center study that included the following places: 1) Outpatient service of the Hospital General de Comalcalco in the state of Tabasco, México, 2) the clinical laboratory “Centro de Investigación Genómica” (Center for Genomic Research) in Comalcalco, Tabasco, México, and c) Universidad Juárez Autónoma de Tabasco (UJAT), specifically the División Académica Multidisciplinaria de Comalcalco (DAMC) in Tabasco, México. Patients were not chosen at random, rather they were recruited from the external consultation of the hospital or from relatives of students from the Faculty of Medicine. Comalcalco is a city in Tabasco with a population of 200 000 inhabitants.

### Ethics Statement

All subjects signed an informed consent to participate in the study after they were given a verbal and written explanation of the research objectives; they did not receive any economical remuneration. Only Mexican subjects descending from Mexican parents and grandparents participated in this study. This study complied with the principles convened in the Helsinki Declaration. In addition, this study was approved by the DAMC-UJAT Ethics and Research Committee (P.O.A. 20111282).

### Data Collection

Persons with type 2 diabetes, previously diagnosed by a physician, were included in this study. Age range was 18–80 years old. Personal and familiar history of diabetes was collected using structured interviews designed specifically for this study. All patients were evaluated and privately interviewed by psychologists or nurses participating in this study. Both psychologists and nurses hold at least a Master’s degree.

### Anthropometric Measurements

In this study the following parameters were collected: height, weight and blood pressure. Height was measured using stadiometers having 1 mm precision and body weight (Kg) using a digital scale with 100-g precision.

Overweight and obesity were classified according to international obesity task force criteria, based on body mass index (BMI) measurements, with cutoff values based on BMI. Cutoff points are a projection of the criteria proposed by WHO for diagnosing overweight (BMI between 25 and 29.9) and obesity (BMI of 30 or more).

To determine arterial blood pressure (BP) we used a calibrated sphygmomanometer, aneroid type, and a stethoscope. Patient was asked to remain seated for ten minutes before the pressure was taken. All the measurements were carried out on left and right arms in compliance with the Mexican official norm NOM-030-SSA2-1999, which refers to Prevention, Treatment and Control of Arterial Hypertension. Measurements were taken by trained and standardized personnel using standard procedures.

### Definition of Diabetes

This study included only patients previously diagnosed diabetes mellitus by a physician. The presence of diabetes was defined as a fasting plasma glucose value ≥7.0 mmol/l (126 mg/dl); individuals with unknown or pre-diabetes status were not included in this study.

### Definition of Complications in Diabetes

Complications in diabetes were considered when patients presented retinopathy, cardiomyopathies, nephropathies, and neuropathies associated with diabetes mellitus.

### Definition of Depression and Anxiety

We evaluated depression and anxiety with the Hamilton Depression Rating Scale (Ham-D) and the Hamilton Anxiety Rating Scale (Ham-A), respectively [33]. Both scales are commonly used at the health service of the Hospital de Comalcalco in Tabasco.

Ham-D is the most commonly used observer-rated depressive symptom rating scale. Although the original scale has 21 items, we used a 17-item reduced version. Nine items with quantifiable severity were ranked on a scale 0–4 and those measuring symptoms that are difficult to assess reliably were ranked on a scale 0–2 (8 items) [34]. The range of the 17-item scale was 0–50, with 14 considered to be the cutoff point of this scale; higher scores indicate more severe depression.

The Ham-A is a 14-item clinician-rated instrument designed to assess and quantify the severity of anxiety. Each item is rated on a five-point Likert-type scale ranging from 0 to 4. Although the scale assesses a broad range of symptoms, it is most frequently used to assess the severity of general anxiety disorder [35], [36]. Ham-A is comprised of psychic and somatic subscales. The psychic subscale (items 1–6 and 14) evaluates the more subjective cognitive and affective complaints of anxiety (e.g., anxious mood, tension, fears, difficulty concentrating); it is particularly useful in assessing the severity of general anxiety disorder. The somatic component (items 7–13) emphasizes features of general anxiety disorder such as autonomic arousal, as well as respiratory, gastrointestinal and cardiovascular symptoms.

### Statistical Analysis

All data are presented as numbers (in percentage) for categorical variables. Ratios were calculated for the variables of interest. Descriptive statistics was used to characterize the sample. The chi-squared test was conducted to compare variables. Multivariate analysis using multiple logistic regressions was carried out to evaluate the combined effect of several factors associated with anxiety and depression among persons with type 2 diabetes after adjusting for confounding variables. Results are presented as adjusted odds ratios (AOR) with 95% CI, which express the magnitude of the effect of each category on the outcome relative to the reference category.
